# Supplementary material for: Affective and social pain modulation in children—Experimental evidence using picture viewing
Source: PLoS One. 2024 Dec 19;19(12):e0313636. doi: 10.1371/journal.pone.0313636 (PMC11658489; doi:10.1371/journal.pone.0313636)
Supplement: S1 Table — Ratings of 12 pictures of an internet search by 8 to 14-year-old children in school, class by class. Self-assessment manikins were used for valence and arousal ratings (SAM; [41]. Pictures were presented for 6 s each in different picture sets on a screen in front of the class. (DOCX) [file pone.0313636.s001.docx]

**S1 Table. Valence and arousal ratings of pictures from an internet search.**

| **Content** | **Picture number** | **Valence rating (SAM 1-9)** | | |  | **Arousal rating (SAM 1-9)** | | |
| --- | --- | --- | --- | --- | --- | --- | --- | --- |
|  |  | **N** | **M** | **SD** |  | **N** | **M** | **SD** |
| Fire works | 21 | 555 | 7.97 | 1.33 |  | 554 | 4.81 | 2.72 |
| Ferrari | 25 | 556 | 7.25 | 2.08 |  | 554 | 4.30 | 3.00 |
| Wildwater rafting | 26 | 433 | 7.65 | 1.67 |  | 434 | 4.55 | 2.81 |
| Seven dolphins | 27 | 147 | 8.33 | 1.23 |  | 147 | 4.41 | 2.94 |
| Huskies in the snow | 35 | 432 | 8.06 | 1.40 |  | 431 | 4.59 | 2.81 |
| Space shuttle | 38 | 555 | 7.03 | 1.99 |  | 553 | 5.37 | 2.96 |
| Dragon roller coaster | 39 | 430 | 7.57 | 1.83 |  | 428 | 5.04 | 2.82 |
| Orca | 40 | 432 | 7.62 | 1.69 |  | 431 | 4.67 | 2.82 |
| Three dolphins springing | 46 | 407 | 8.27 | 1.36 |  | 407 | 4.79 | 2.99 |
| Diving tower | 49 | 407 | 6.78 | 2.13 |  | 406 | 4.75 | 2.74 |
| Chairoplane | 50 | 406 | 7.27 | 1.95 |  | 407 | 4.56 | 2.72 |
| Two orcas springing | 51 | 408 | 7.96 | 1.58 |  | 407 | 5.15 | 2.94 |

Ratings of 12 pictures of an internet search by 8 to 14-year-old children in school, class by class. Self-assessment manikins were used for valence and arousal ratings (SAM; Bradley und Lang 1994). Pictures were presented for 6 s each in different picture sets on a screen in front of the class.

**Reference**

Bradley, Margaret M., Lang, Peter J. (1994): Measuring emotion: the self-assessment manikin and the semantic differential. In: *Journal of Behavior Therapy and Experimental Psychiatry* 25 (1), S. 49–59. DOI: 10.1016/0005-7916(94)90063-9.
