# Supplementary material for: Affective and social pain modulation in children—Experimental evidence using picture viewing
Source: PLoS One. 2024 Dec 19;19(12):e0313636. doi: 10.1371/journal.pone.0313636 (PMC11658489; doi:10.1371/journal.pone.0313636)
Supplement: S2 Table — Means and standard deviations (SD) for experimental pain measures, affective, and social pain modulation for the total sample as well as for healthy children and children with recurrent pain separately and the comparison between the two latter groups. (DOCX) [file pone.0313636.s002.docx]

**S2 Table. Data of experimental pain measures and pain modulation.**

|  |  | **Total sample**  **(n = 42)** | |  | | **Healthy children**  **(n = 27)** | |  | | **Children with recurrent pain (n = 15)** | |  | | **Healthy vs recurrent pain** | |
| --- | --- | --- | --- | --- | --- | --- | --- | --- | --- | --- | --- | --- | --- | --- | --- |
|  |  | | ***Mean (SD)*** | |  | | ***Mean (SD)*** | |  | | ***Mean (SD)*** | |  | | ***t (df), p*** |
| *Experimental pain measures* | | | | | | | | | | | | | | | |
| Heat pain threshold (°C) |  | | 41.32 (3.29) | |  | | 41.43 (3.22) | |  | | 41.12 (3.53) | |  | | 0.29 (40), .777 |
| Experimental temperature (°C) |  | | 42.10 (2.94) | |  | | 42.26 (2.79) | |  | | 41.80 (3.27) | |  | | 0.48 (40), .633 |
| VAS t1 |  | | 8.04 (3.76) | |  | | 7.74 (3.60) | |  | | 8.57 (4.12) | |  | | -0.69 (40), .497 |
| *Pain modulation* | | | | | | | | | | | | | | | |
| PosPM t2 |  | | 0.90 (2.18) | |  | | 1.01 (2.20) | |  | | 0.69 (2.21) | |  | | 0.46 (40), .650 |
| PosPM t3 |  | | 0.91 (1.81) | |  | | 0.67 (1.67) | |  | | 1.36 (2.09) | |  | | -1.17 (40), .248 |
| NegPM t2 |  | | 0.37 (2.65) | |  | | 0.46 (2.41) | |  | | 0.22 (3.11) | |  | | 0.27 (40), .787 |
| NegPM t3 |  | | 0.87 (2.58) | |  | | 1.12 (2.95) | |  | | 0.42 (1.73) | |  | | 0.84 (40), .405 |
| MotherPM t2 |  | | 1.22 (2.69) | |  | | 0.79 (1.69) | |  | | 2.00 (3.85) | |  | | -1.42 (17^1^), .263 |
| MotherPM t3 |  | | 0.73 (2.65) | |  | | 0.35 (1.87) | |  | | 1.42 (3.64) | |  | | -1.27 (40), .211 |
| HappyPM t2 |  | | 0.61 (1.81) | |  | | 0.57 (1,68) | |  | | 0.69 (2.09) | |  | | -0.21 (40), .839 |
| HappyPM t3 |  | | 0.56 (1.66) | |  | | 0.37 (1.53) | |  | | 0.91 (1.89) | |  | | -1,01 (40), .319 |

Means and standard deviations (SD) for experimental pain measures, affective, and social pain modulation for the total sample as well as for healthy children and children with recurrent pain separately and the comparison between the two latter groups.

^1^corrected for significant differences in variance (Levene’s Test for Equality of Variances: *F* = 6.49, *p* = .015).

Abbreviations: VAS: Visual analogue scale; t1 = before picture presentation, t2 = after pictures 1-3, t3 = after pictures 4-6; posPM = positive pain modulation: refers to the difference when viewing neutral strangers’ faces versus positive scenes, posPM > 0 indicates a pain relieving effect of positive scenes; negPM = refers to the difference when viewing negative scenes versus neutral strangers’ faces, negPM > 0 indicates a pain enhancing effect of negative scenes; motherPM = mothers’ faces pain modulation: refers to the difference when viewing neutral strangers’ faces versus neutral mothers’ faces, motherPM > 0 indicates a pain relieving effect of mother faces; happyPM = happy faces pain modulation: refers to the difference when viewing neutral strangers’ faces versus happy strangers’ faces, motherPM > 0 indicates a pain relieving effect of mother faces.
